# COVID-19 antibody seroprevalence in residential psychiatric inpatients

**DOI:** 10.1192/bjo.2021.305

**Published:** 2021-06-18

**Authors:** Sheena Shah, Arshad Hussain, Sabreena Qadri, Fazle Roub, Insha Rauf, Praveen Kumar

**Affiliations:** 1 Institute of Mental Health and Neurosciences; 2 NHS Scotland

## Abstract

**Aims:**

While other mental health care outpatient facilities were moved to COVID-centers in March 2020 during the COVID-19 pandemic, the Institute of Mental Health and Neurosciences in Kashmir remained the only functional outpatient facility in the region. It is the only mental health care hospital in the country with a residential facility for psychiatric inpatients catering to the whole population of Jammu and Kashmir, India. The Mental Health Care Act 2017 that neccesitated “halfway homes” is yet to be implemented in the state leaving it's inpatients entirely under the institution's care. This study is to investigate the seroprevalence of antibodies to SARS-COVID-19 virus in the 34 residential inpatients in separate male (23 patients) and female (11 patients) wards. This was done as an audit to strategies and measures taken by the institute in protecting it's inpatients.

**Method:**

3 to 5 ml of peripheral venous blood samples were collected and plasma extracted and analysed using the CE-IVD Roche Cobas Elecsys AntiSARS-CoV-2, Electrochemiluminescence Immunoassay (ECLIA) for the qualitative detection of total Immunoglobulins (IgG, IgM and IgA; Pan Ig) generated against SARS-CoV-2 (Roche Diagnostics, Indianapolis, IN, USA). The test was performed according to the manufacturer's instructions.

**Result:**

Out of the 34 inpatients, 2 male inpatients tested positive for antibodies against SARS-CoV-2 (seroprevalence of 5.88%). In comparison, based on a report conducted by the government's Department of Community Medicine and Biochemistry on the 28th of October 2020, out of 2,361 participants in the community, 959 tested positive (seroprevalence of 40.6%).

One of the inpatients that tested positive was re-admitted after testing negative via RT-PCR. The second patient was admitted after being found homeless. He was tested negative on day 1 via RAT and on day 5 via RT-PCR. We believe both of them aquired the infection in the community.

**Conclusion:**

This audit shows that the strategies implemented by the institute were effective in the prevention of the spread of COVID-19. Practical implementations of what works and improvisations are the proven methods of decreasing the mortality and morbidity in vulnerable populations while continuously providing vital mental health services.

